# Synthesis of Chromen-4-One-Oxadiazole Substituted Analogs as Potent *β*-Glucuronidase Inhibitors

**DOI:** 10.3390/molecules24081528

**Published:** 2019-04-18

**Authors:** Muhammad Taha, Fazal Rahim, Muhammad Ali, Muhammad Naseem Khan, Mohammed A. Alqahtani, Yasser A. Bamarouf, Mohammed Gollapalli, Rai Khalid Farooq, Syed Adnan Ali Shah, Qamar Uddin Ahmed, Zainul Amiruddin Zakaria

**Affiliations:** 1 Department of Clinical Pharmacy, Institute for Research and Medical Consultations (IRMC), Imam Abdulrahman Bin Faisal University, P.O. Box 1982, Dammam 31441, Saudi Arabia; 2 Department of Chemistry, Hazara University, Mansehra 21300, Khyber Pakhtunkhwa, Pakistan; fazalstar@gmail.com; 3 Natural and Medical Sciences Research Center, University of Nizwa, P.O. Box 33, Birkat Al Mauz, Nizwa 616, Sultanate of Oman; m_alisaad@yahoo.com; 4 Department of Chemistry, COMSATS Institute of Information Technology, University Road, Abbottabad 22060, KPK, Pakistan; naseemkhan.tareen@gmail.com; 5 Department of Computer Information Systems, College of Computer Science & Information Technology, Imam Abdulrahman Bin Faisal University, P.O. Box 1982, Dammam 31441, Saudi Arabia; maqhtani@iau.edu.sa (M.A.A.); yabamarouf@iau.edu.sa (Y.A.B.); magollapalli@iau.edu.sa (M.G.); 6 Department of Neuroscience Research, Institute for Research and Medical Consultations (IRMC), Imam Abdulrahman Bin Faisal University, P.O. Box 1982, Dammam 3144, Saudi Arabia; rkfarooq@iau.edu.sa; 7 Faculty of Pharmacy, Universiti Teknologi MARA Puncak Alam Campus, 42300 Bandar Puncak Alam, Selangor D.E., Malaysia; benzene301@yahoo.com; 8 Atta-ur-Rahman Institute for Natural Products Discovery (AuRIns), Universiti Teknologi MARA Puncak Alam Campus, 42300 Bandar Puncak Alam, Selangor D.E., Malaysia; 9 Department of Pharmaceutical Chemistry, Kulliyyah of Pharmacy, International Islamic University Malaysia, 25200 Kuantan, Pahang DM, Malaysia; quahmed@iium.edu.my; 10 Department of Biomedical Science, Faculty of Medicine and Health Sciences, Universiti Putra Malaysia, 43400 Serdang, Selangor, Malaysia; 11 Halal Institute Research Institute, Universiti Putra Malaysia, 43400 Serdang, Selangor, Malaysia

**Keywords:** chromen-4-one, oxadiazole, synthesis, *β*-glucuronidase inhibition, molecular docking, SAR

## Abstract

Chromen-4-one substituted oxadiazole analogs **1**–**19** have been synthesized, characterized and evaluated for *β*-glucuronidase inhibition. All analogs exhibited a variable degree of *β*-glucuronidase inhibitory activity with IC_50_ values ranging in between 0.8 ± 0.1–42.3 ± 0.8 μM when compared with the standard d-saccharic acid 1,4 lactone (IC_50_ = 48.1 ± 1.2 μM). Structure activity relationship has been established for all compounds. Molecular docking studies were performed to predict the binding interaction of the compounds with the active site of enzyme.

## 1. Introduction

*β*-Glucuronidase (E.C.3.2.1.31) is one of the most extensively studied enzymes in the metabolic hydrolysis of conjugated compounds, and eliminates large number of toxic compounds from body as glucuronides [1]. It acts as a glycoside hydrolase enzyme which induces to breaking of glucuronosyl-O-bonds [2]. In human body *β*-glucuronidase is present in many body fluids and organs [3]. Increased activity of *β*-glucuronidase has resulted in various health issues that include renal disorders [4], urinary tract infections [5], epilepsy [6], renal transplant rejection [7], neoplasm of bladder [8] and breast cancers [9]. Bacterial *β*-glucuronidase and *β*-glucosidase in the human colon are involved in the metabolism and activation of xenobiotics derived from dietary compounds [10]. The enzymes are identified to be mediators of colon cancer (CRC). Many carcinogenic dietary compounds are metabolized in the liver and then conjugated to glucuronic acid before being excreted with bile into the small intestine. In the colon, bacterial *β*-glucuronidase hydrolyzes the conjugates, there by releasing the parent compounds or their activated hepatic metabolites [11]. Epidemiological studies have shown that populations at high risk for colorectal cancer have high levels of fecal *β*-glucuronidase activity and CRC patients have significantly higher levels of the enzyme than healthy controls [12].

Chromone (1,4-benzopyrone) is a derivative of benzopyran with a substituted keto group on the pyran ring. Chromones (4H-chromen-4-ones) constitute a well-known class of naturally occurring oxygen-containing heterocycles. These compounds exhibit important biological properties such as anticancer [13], cytotoxic [14], antioxidant [15], anti-inflammatory [16], antifungal activities [17].

1,3,4-Oxadiazole containing compounds have remarkable biological and pharmacological properties. Recently, differently substituted 1,3,4-oxadiazoles have been reported for their biological activities such as antibacterial [18], antifungal [19], insecticidal [20], antiviral [21], inflammatory [22], antitubercular [23], antitumor [24] and analgesic activity [25].

Our research group has been working on synthesis of variety of heterocycles and explored a huge biological potential of these compounds [26,27,28,29,30,31,32]. Present work belongs to a simple and reliable synthetic protocol that has been developed for the synthesis of a series of chromone substituted oxadiazole pharmacophores in another effort towards developing more novel agents against *β*-glucuronidase enzyme. This is beyond any doubt that combination of two or more biologically active compounds grants newer molecules with complementary pharmacophoric potentials or with different mechanisms of actions. The unity of pharmacophoric approach, as a result, could possibly address the active site of different targets more effectively and could offer the possibility to overcome the issue of drug resistance and unwanted effects [33,34,35,36]. The outcomes of this merger have already been the subject of attention in finding new antimicrobials [37,38,39,40,41].

## 2. Result and Discussions

### 2.1. Chemistry

**Scheme 1:**
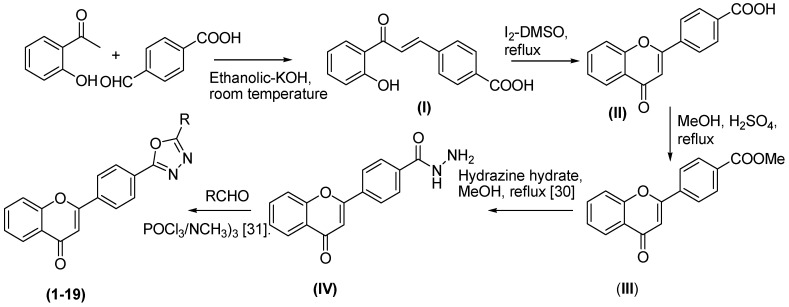
Synthesis of chromene based oxadiazole derivatives **1**–**19**.

The synthesis of the novel series of chromene-based oxadiazole *β*-glucuronidase inhibitors **1–19** commenced with the reaction of 2-hydroxyacetophenone and *p*-formylbenzoic acid in ethanolic potassium hydroxide at room temperature (Scheme 1).

**Figure 1:**
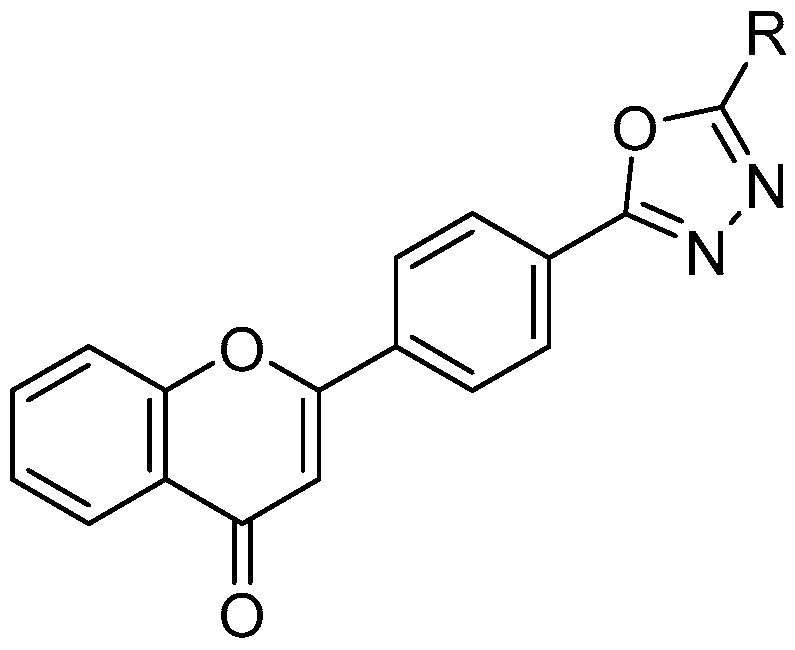
Structure of the basic skeleton of the chromen-4-one based oxadiazole derivatives **1**–**19**.

The adduct **I** underwent oxidative cyclization to form 4-substituted chromenone analog **II**. This acid analog **II** was esterified to give **III** and reacted with hydrazine hydrate to form the corresponding hydrazide **IV** which upon reaction with the corresponding aldehydes under the described conditions formed the desired oxadiazoles **1**–19 Figure 1. All synthesized compounds were characterized using various spectroscopic methods.

### 2.2. β-Glucuronidase Activity

4-Substituted chromenone-based oxadiazole derivatives **1**–**19** were synthesized and evaluated for *β*-glucuronidase inhibitory potential. All the synthesized analogs showed potent inhibitory potential, ranging between 0.8 ± 0.1 µM to 42.3 ± 0.8 µM when compared with the standard d-saccharic acid 1,4 lactone (IC_50_ value = 48.1 ± 1.2 µM) (Table 1). The structure activity relationship has been established on the basis of substitution pattern on the phenyl ring.

**Figure 2:**
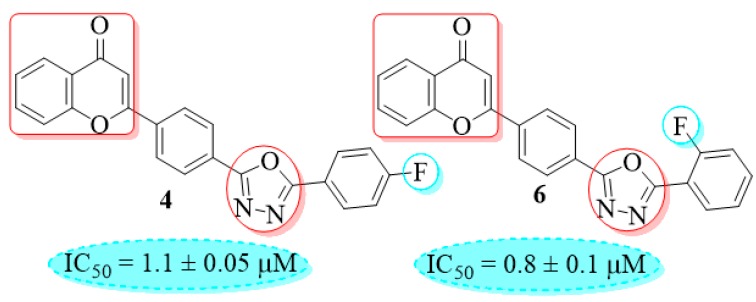
*ortho*-Fluoro and *para*-fluoro analogs (most potent ones).

The compound **6** (IC_50_ = 0.8 ± 0.1 μM), an *ortho*-fluoro analog was found to be the most potent among the series. The greater potential shown by this analog might be due to the electron withdrawing fluoro group. If we compare it with other fluoro analogs like compound **4** (IC_50_ = 1.1 ± 0.05 μM) a *para*-fluoro analog and compound **5** (IC_50_ = 3.8 ± 0.2 μM), a *meta*-fluoro analog, the analog **6** was found superior than the analogs **4** and **5**. The little bit difference in their potential is appears to be due to the difference in the position of fluoro groups on phenyl ring Figure 2.

The compound **3,** an *ortho*-chloro analog (IC_50_= 2.1 ± 0.1 μM) was found to be the second most potent analog among the series if we compare it with other chloro analogs like compound **1** (IC_50_ = 2.6 ± 0.1 μM) a *para*-chloro analog and compound **2** (IC_50_ = 5.1 ± 0.2 μM) a *meta*-flouro analog. The greater potential is again seemingly due to position of substituent(s) on phenyl ring.

Similarly, compound **9**, an *ortho* methyl analog (IC_50_= 8.6 ± 0.3 μM) showed outstanding inhibitory potential. If we compare it with other methyl analogs like analog **7** (IC_50_ = 9.4 ± 0.3 μM) a *para*-methyl analog and compound **8** (IC_50_ = 13.0 ± 0.3 μM) a *meta*-methyl analog, compound **9** is superior.

Similarly compound **12** (IC_50_ = 17.2 ± 0.4 μM), an *ortho*-nitro analog showed greater potential if we compare it with other nitro analogs like compound **11** (IC_50_ = 26.1 ± 0.5 μM) a *meta*-nitro analog and compound **10** (IC_50_ = 21.3 ± 0.4 μM) a *para*-nitro analog. The same pattern was observed in other analogs with different substituents.

A closer look upon the structures of compounds and their corresponding IC_50_ values revealed that the presence of different substitutions i.e., fluoro. chloro, methyl, nitro, methoxy and pyridinyl on the aromatic side chain is of great importance. It was also observed that either an EWG or EDG on the phenyl part showed great potential but the slight difference in potential was mainly affected by the position of the substituent.

Further to above, it is evident from the results that the presence of halogens or alkyl groups on benzene ring influenced the inhibitory potentials significantly. Similarly, nitro substitutions reduced the potential of inhibition. Apart from direct interactions of these attachments, it can be easily argued that these substitutions significantly affected the electronic distributions and intensities at the active sites. In many cases, oxadiazole part also played a significant role in forming hydrogen bond interactions with the active site of enzyme. Most important of these positions are Asp 207, Glu 451 and Glu 540. To better understand the binding interactions of the most active analogs, molecular docking analysis were performed.

### 2.3. Molecular Docking Studies

Experimental results were further validated by carrying out molecular docking studies and more information was obtained concerning binding modes of synthesized compounds within the active site of *β*-Glucuronidase. The X-ray structure of *β*-glucuronidase was retrieved from the Protein Data Bank with PDB ID: 1BHG. All synthesized compounds were docked into the active site of protein and conformations selected for each docked compound were of least energy and were pictured to further understand the interactions between ligands and receptor.

**Figure 3:**
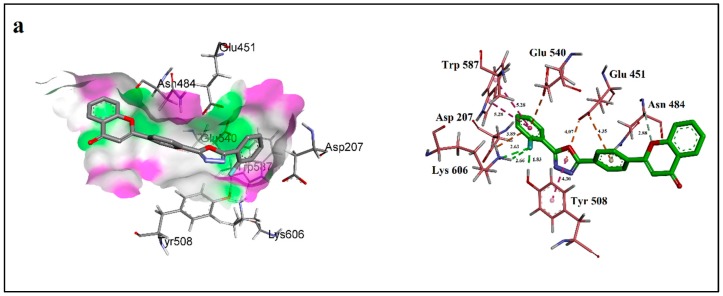

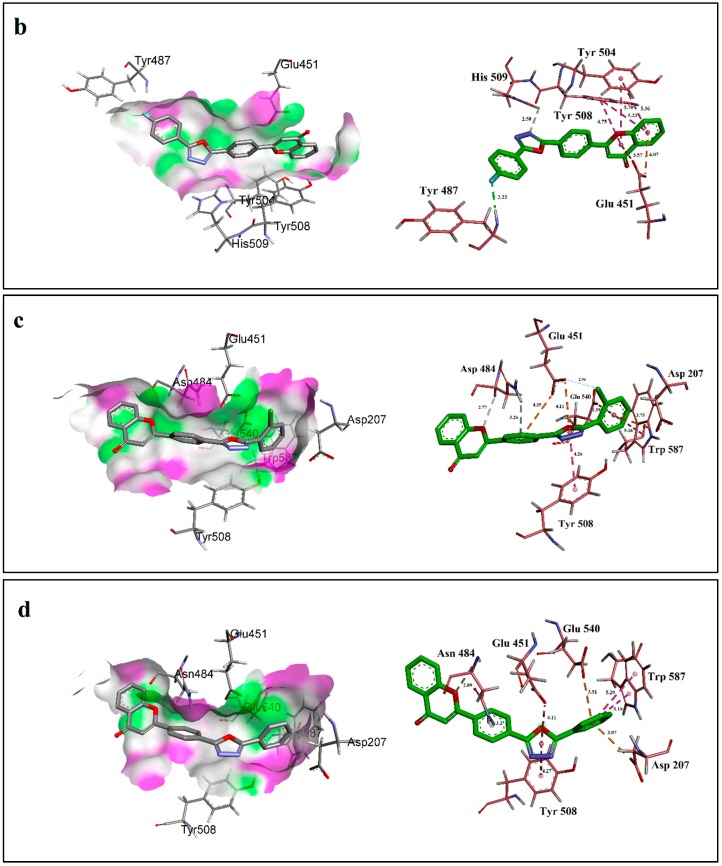
Ligand–protein interactions of compound (**a**) 6, (**b**) 4, (**c**) 3 and (**d**) 19 with the active site of *β*-glucuronidase (1BHG) created by using Discovery Studio 17.2. The left side displays 3D maps of the hydrogen bonds of the compounds in the binding site. The right side shows the 2D interaction patterns. Dashed lines show the interactions among the ligand and the amino acids of the protein. Distances are in Angstrom.

Binding mode of compound **6**, the most active member (IC_50_ = 0.80 ± 0.10 µM) among the series was analyzed. Three conventional hydrogen bond interactions were observed between the fluorine atom at *ortho* position and side chain amino acids including Tyr 508 and Lys 606 with a internuclear distance of 2.63 Å, 2.66 Å and 1.83 Å respectively. Meanwhile, C-H and N-H groups of Asn 484 were involved in forming carbon hydrogen bond with ether oxygen of chromene-4-one ring (2.88 Å) and π-donor hydrogen bond with the central arene ring of compound **6** (3.14 Å). Significant residues like Asp 207, Glu 451 and Glu 540 were not involved in hydrogen bonding due to absence of hydrogen bond donor groups on compound **6** instead, these residues were involved in π-anion interactions as shown in Figure 3a. Furthermore, the ligand /receptor complex was further stabilized by π-π T-shaped and π-π Stacked interactions between indole ring of Trp 587 and phenol ring of Tyr 508 respectively.

Compound **4**, the second most active member with IC_50_ = 1.10 ± 0.05 µM was evaluated and a conventional hydrogen bond between fluorine atom at *para* position and N-H group of Tyr 487 with a internuclear distance of 2.22 Å and carbon hydrogen bond between nitrogen atom of pyrazole ring of compound **4** and C-H (at imidazole ring) of His 509 (2.58 Å) were detected. Meanwhile, the catalytic residue Glu 451, as in case of compound **6** was also involved in forming two π-Anion interactions with the chromene-4-one ring of compound **4**. Side chain residues involved in π-π T-Shaped interactions with compound **4** were Tyr 504 and Tyr 508 as shown in Figure 3b.

Analysis of interactions between third most active compound **3** (IC_50_ = 2.10 ± 0.10 µM) and active site of *β*-Glucuronidase revealed that it was not involved in conventional hydrogen bonding. It was found that the ether oxygen atom of chromene-4-one ring and central arene ring of compound **3** were involved in forming carbon hydrogen bond (2.77 Å) and π-Donor hydrogen bond (3.26 Å) with the C-H and N-H groups of Asn 484 respectively. The chlorine atom presents at *ortho* position formed so-called halogen bond of distance 2.94 Å and bond angle ~153° with the carboxyl oxygen of Glu 451. Other interactions shown by compound 3 were almost the same as those shown by compound 6 (Figure 3c).

The least active compound **19** (IC_50_ = 42.30 ± 0.80 µM) among the series failed to form many interactions as those made by active compounds including conventional hydrogen bonding and halogen bonding. It was involved in forming carbon hydrogen and π-Donor hydrogen bond with Asn 484 as formed by compound **3** but with one longer bond distance i.e., 2.89 Å and 3.2 Å. Other interactions resulted between compound **19** and Asp 207, Glu 451, Tyr 508, Glu 540, Trp 587 of the side chain were π-Anion (3.87 Å), π-Anion (4.11 Å), π-π Stacked (4.27 Å), π-Anion (3.51) and π-π T-shaped (5.19 Å and 5.29 Å) respectively (Figure 3d).

## 3. Conclusions

Chromen-4-one based oxadiazole derivatives **1**–**19** have been synthesized and evaluated for *β*-glucuronidase inhibitory potential. All analogs displayed potent *β*-glucuronidase inhibitory potential ranging between 0.8 ± 0.1 to 42.3 ± 0.8 µM as a compare to standard d-saccharic acid 1,4 lactone (IC_50_ = 48.1 ± 1.2μM). The fluoro derivatives were found to be the more potent among the series. A molecular docking study indicated that the top ranked conformations of almost all compounds were well accommodated inside the active site of *β*-glucuronidase enzyme and they were involved in various types of interactions with the active site residues of the *β*-glucuronidase enzyme.

## 4. Experimental

### 4.1. General Information

The intermediates 4-(4-oxo-4*H*-chromen-2-yl)benzoic acid (**II**), methyl 4-(4-oxo-4*H*-chromen-2-yl)benzoate (**III**) and 4-(4-oxo-4*H*-chromen-2-yl)benzohydrazide (**IV**) were synthesized as reported in our previous paper [42].

### 4.2. General Procedure for Synthesis of Flavone-Based Oxadiazoles

Compound **IV** (0.5 mmol) and a substituted arylcarboxylic acid (0.5 mmol) were mixed in a 50 mL round bottom flask then POCl_3_ (5 mL) was added dropwise [33]. The mixture was refluxed for 4–5 h while the reaction progress was monitored using TLC until completion, when the reaction mixture cooled to room temperature and poured onto crushed ice. NaHCO_3_ solution was added and the resulting solid mass that precipitated out was filtered, dried, and recrystallized from methanol in good to excellent yields.

### 4.3. 2-(4-(5-(4-Chlorophenyl)-1,3,4-oxadiazol-2-yl)phenyl)-4H-chromen-4-one (***1***)

Solid; Yield 75%; m.p. 288–289 °C; ^1^H-NMR (MeOD-*d*_4_, 500 MHz): *δ* 8.05 (d, *J* = 8.5 Hz, 2H), 7.96 (d, *J* = 8.5 Hz, 2H), 7.84 (d, *J* = 8.0 Hz, 2H), 7.75 (dd, *J* = 8.0, 2.0 Hz, 1H), 7.44 (d, *J* = 8.0 Hz, 2H), 7.20 (s, 1H), 7.17 (td, *J* = 8.5 Hz, 1.0 Hz, 1H), 6.94–6.91 (m, 2H); ^13^C-NMR (125 MHz, DMSO-*d*_6_): *δ* 177.4, 164.5, 164.4, 163.6, 156.2, 135.2, 132.6, 132.2, 132.2, 131.6, 130.1, 128.5, 128.5, 127.8, 127.8, 127.1, 127.1, 126.0, 125.1, 124.0, 123.2, 116.0, 104.6; HR-EI-MS: *m*/*z* Calcd for C_23_H_13_ClN_2_O_3_, [M]^+^ 414.0770; found 414.0752.

### 4.4. 2-(4-(5-(3-Chlorophenyl)-1,3,4-oxadiazol-2-yl)phenyl)-4H-chromen-4-one (***2***)

Solid; Yield 79%; m.p. 282–283 °C; ^1^H-NMR (DMSO-*d*_6_, 500 MHz): *δ* 8.04 (s, 4H), 7.83 (s, 1H), 7.72–7.70 (m, 2H), 7.47 (d, *J* = 4.5 Hz, 2H), 7.31 (s, 1H), 7.16 (t, *J* = 8.5 Hz, 1H), 6.94 (d, *J* = 8.5 Hz, 1H), 6.85 (t, *J* = 8.0 Hz, 1H); ^13^C-NMR (125 MHz, DMSO-*d*_6_): *δ* 177.3, 166.2, 164.4, 156.1, 137.8, 135.1, 134.1, 130.1, 129.9, 129.8, 127.6, 127.6, 127.2, 127.2, 127.0, 126.1, 125.5, 125.5, 125.2, 123.6, 124.2, 116.3, 104.6; HR-EI-MS: *m*/*z* Calcd for C_23_H_13_ClN_2_O_3_, [M]^+^ 414.0771; found 414.0751.

### 4.5. 2-(4-(5-(2-Chlorobenzyl)-1,3,4-oxadiazol-2-yl)phenyl)-4H-chromen-4-one (***3***)

Solid; Yield 81%; m.p. 276–277 °C; ^1^H-NMR (DMSO-*d*_6_, 500 MHz): *δ* ^1^H-NMR (MeOD-d_4_, 500 MHz): *δ* 8.24 (dd, *J* = 8.0, 2.0 Hz, 1H), 8.02 (d, *J* = 8.5 Hz, 2H), 7.96 (d, *J* = 8.5 Hz, 2H), 7.70 (dd, *J* = 8.0, 2.0 Hz, 1H), 7.42 (d, *J* = 8.5 Hz, 1H), 7.43–7.40 (m, 2H), 7.22–7.18 (m, 2H), 6.94–6.91 (m, 2H); ^13^C-NMR (125 MHz, DMSO-*d*_6_): *δ* 177.4, 166.3, 164.1, 156.0, 138.0, 135.3, 134.5, 130.8, 130.6, 129.1, 127.5, 127.5, 127.1, 127.1, 127.0, 126.9, 126.0, 125.2, 124.2, 123.7,123.2, 116.2, 104.7; HR-EI-MS: *m*/*z* Calcd for C_23_H_13_ClN_2_O_3_, [M]^+^ 414.0772; found 414.0754.

### 4.6. 2-(4-(5-(4-Fluorophenyl)-1,3,4-oxadiazol-2-yl)phenyl)-4H-chromen-4-one (***4***)

Solid; Yield 82%; m.p. 299–300 °C; ^1^H-NMR (DMSO-*d*_6_, 500 MHz): *δ* 8.00–7.96 (m, 4H), 7.78–7.75 (m, 3H), 7.33–7.30 (m, 3H), 7.18 (t, *J* = 7.0 Hz, 1H), 6.94 (d, *J* = 8.0 Hz, 1H), 6.88 (t, *J* = 7.0 Hz, 1H); ^13^C-NMR (125 MHz, DMSO-*d*_6_): *δ* 177.8, 164.6, 164.6, 163.8, 163.0, 156.1, 135.0, 130.5, 129.3, 129.3, 127.6, 127.6, 126.8, 126.8, 125.6, 125.5, 124.1, 123.6, 121.5, 116.3, 115.8, 115.8, 104.7; HR-EI-MS: *m*/*z* Calcd for C_23_H_13_FN_2_O_3_, [M]^+^ 384.0910; found 384.0894.

### 4.7. 2-(4-(5-(3-Fluorophenyl)-1,3,4-oxadiazol-2-yl)phenyl)-4H-chromen-4-one (***5***)

Solid; Yield 80%; m.p. 294–295 °C; ^1^H-NMR (DMSO-*d*_6_, 500 MHz): *δ* 8.02 (d, *J* = 7.5 Hz, 2H), 87.96 (d, *J* = 7.5 Hz, 2H), 7.74–7.71 (m, 2H), 7.60 (d, *J* = 7.0 Hz, 1H), 7.42 (d, *J* = 8.5 Hz, 1H), 7.20–7.17 (m, 3H), 6.96–6.982 (m, 2H); ^13^C-NMR (125 MHz, DMSO-*d*_6_): *δ* 177.6, 164.4, 164.4, 163.5, 162.1, 135.1, 130.4, 128.0, 127.6, 127.5, 127.5, 127.3, 127.3, 127.1, 127.1, 125.9, 124.1, 123.6, 123.0, 116.0, 115.8, 115.3, 104.3; HR-EI-MS: *m*/*z* Calcd for C_23_H_13_FN_2_O_3_, [M]^+^ 384.0911; found 384.0892.

### 4.8. 2-(4-(5-(2-Fluorophenyl)-1,3,4-oxadiazol-2-yl)phenyl)-4H-chromen-4-one (***6***)

Solid; Yield 78%; m.p. 291–292 °C; ^1^H-NMR (MeOD-*d*_4_, 500 MHz): *δ* 8.20 (t, *J* = 7.0 Hz, 1H), 8.03 (d, *J* = 8.0 Hz, 2H), 7.97 (d, *J* = 8.0 Hz, 2H), 7.72 (dd, *J* = 7.0, 20 Hz, 1H), 7.44 (d, *J* = 8.5 Hz, 1H), 7.25 (t, *J* = 8.0 Hz, 1H), 7.22–7.18 (m, 3H), 6.97–6.95 (m, 2H); ^13^C-NMR (125 MHz, DMSO-*d*_6_): *δ* 177.5, 164.4, 164.4, 163.5, 158.2, 156.1, 135.0, 130.1, 129.0, 127.2, 127.2, 126.8, 126.8, 125.6, 125.2, 125.0, 124.9, 123.8, 123.4, 123.3, 116.0, 114.5, 104.4; HR-EI-MS: *m*/*z* Calcd for C_23_H_13_FN_2_O_3_, [M]^+^ 384.0912; found 384.0895.

### 4.9. 2-(4-(5-(p-Tolyl)-1,3,4-oxadiazol-2-yl)phenyl)-4H-chromen-4-one (***7***)

Solid; Yield 83%; m.p. 270–271 °C, ^1^H-NMR (MeOD-*d*_4_, 500 MHz): *δ* 8.03 (d, *J* = 8.0 Hz, 2H), 7.96 (d, *J* = 8.0 Hz, 2H), 7.74–7.71 (m, 3H), 7.25 (d, *J* = 8.0 Hz, 2H), 7.23–7.20 (m, 2H), 6.97–6.95 (m, 2H), 2.43 (s, 3H); ^13^C-NMR (125 MHz, DMSO-*d*_6_): *δ* 177.4, 164.6, 164.6, 163.5, 156.0, 142.1, 135.1, 131.5, 130.2, 127.3, 127.3, 127.2, 127.2, 126.8, 126.8, 126.2, 126.2, 125.6, 125.2, 123.7, 123.5, 116.0, 104.3, 21.2; HR-EI-MS: *m*/*z* Calcd for C_24_H_16_N_2_O_3_, [M]^+^ 380.1161; found 380.1145.

### 4.10. 2-(4-(5-(m-Tolyl)-1,3,4-oxadiazol-2-yl)phenyl)-4H-chromen-4-one (***8***)

Solid; Yield 85%; m.p. 264–265 °C, ^1^H-NMR (DMSO-*d*_6_, 500 MHz): *δ* 8.03 (m, 4H), 7.72(s, 1H), 7.56 (s, 1H), 7.51 (d, *J* = 7.0 Hz, 1H), 7.42 (s, 1H), 7.32 (t, *J* = 7.0 Hz, 1H), 7.24 (d, *J* = 7.0 Hz, 1H), 7.16 (t, *J* = 8.5 Hz, 1H), 6.95(d, *J* = 7.5 Hz, 1H), 6.89 (t, *J* = 7.5 Hz, 1H), 2.39 (s, 3H); ^13^C-NMR (125 MHz, DMSO-*d*_6_): *δ* 177.5, 164.5, 164.5, 163.6, 156.2, 139.0, 135.2, 130.3, 129.1, 129.0, 128.8, 127.3, 127.3, 126.7, 126.7, 126.0, 125.9, 125.2, 124.5, 123.9, 123.2, 116.0, 104.3, 21.5; HR-EI-MS: *m*/*z* Calcd for C_24_H_16_N_2_O_3_, [M]^+^ 380.1160; found 380.1143.

### 4.11. 2-(4-(5-(o-Tolyl)-1,3,4-oxadiazol-2-yl)phenyl)-4H-chromen-4-one (***9***)

Solid; Yield 80%; m.p. 260–261 °C; ^1^H-NMR (DMSO-*d*_6_, 500 MHz): *δ* 8.02–7.97 (m, 5H), 7.31–7.28 (m, 4H), 7.85 (d, *J* = 7.0 Hz, 1H), 7.16 (t, *J* = 7.0 Hz, 1H), 6.94 (s, 1H), 6.88 (t, *J* = 7.0 Hz, 1H), 2.44 (s, 3H); ^13^C-NMR (125 MHz, DMSO-*d*_6_): *δ* 177.3, 164.3, 164.3, 163.5, 156.1, 137.1, 136.8, 135.1, 130.2, 129.4, 128.5, 127.3, 127.3, 127.2, 126.7, 126.7, 126.1, 125.8, 125.2, 123.6, 123.2, 116.0, 104.4, 18.5; HR-EI-MS: *m*/*z* Calcd for C_24_H_16_N_2_O_3_, [M]^+^ 380.1161; found 380.1141.

### 4.12. 2-(4-(5-(4-Nitrophenyl)-1,3,4-oxadiazol-2-yl)phenyl)-4H-chromen-4-one (***10***)

Solid; Yield 85%; m.p. 304–305 °C; ^1^H-NMR (DMSO-*d*_6_, 500 MHz): *δ* 8.29–8.25 (m, 2H), 8.03–7.99 (m, 6H), 7.72 (d, *J* = 7.5 Hz, 1H), 7.34 (s, 1H), 7.17 (t, *J* = 7.0 Hz, 1H), 6.94 (d, *J* = 8.0 Hz, 1H), 6.89 (t, *J* = 7.0 Hz, 1H); ^13^C-NMR (125 MHz, DMSO-*d*_6_): *δ* 177.4, 164.4, 164.4, 163.4, 156.0, 147.8, 135.1, 132.0, 131.0, 131.0, 130.2, 128.6, 128.6, 127.2, 127.2, 127.0, 127.0, 125.6, 125.2, 123.7, 123.3, 116.0, 104.5; HR-EI-MS: *m*/*z* Calcd for C_23_H_13_N_3_O_5_, [M]^+^ 411.0855; found 411.0839.

### 4.13. 2-(4-(5-(3-Nitrophenyl)-1,3,4-oxadiazol-2-yl)phenyl)-4H-chromen-4-one (***11***)

Solid; Yield 82%; m.p. 296–297 °C; ^1^H-NMR (DMSO-*d*_6_, 500 MHz): *δ* 8.56–8.52 (m, 2H), 8.27(d, *J* = 7.0 Hz, 1H), 8.14 (d, *J* = 7.0 Hz, 1H), 8.03–7.98 (m, 3H), 7.74–7.71 (m, 2H), 7.35 (s, 1H), 7.15 (t, *J* = 7.5 Hz, 1H), 6.94 (d, *J* = 7.0 Hz, 1H), 6.88 (t, *J* = 7.0 Hz, 1H); ^13^C-NMR (125 MHz, DMSO-*d*_6_): *δ* 177.5, 164.4, 164.4, 163.4, 156.0, 148.3, 135.1, 133.8, 130.2, 130.1, 127.4, 127.4, 127.0, 126.8, 126.8, 125.8, 125.2, 123.8, 123.8, 123.2, 122.6, 116.0, 104.4; HR-EI-MS: *m*/*z* Calcd for C_23_H_13_N_3_O_5_, [M]^+^ 411.0855; found 411.0837.

### 4.14. 2-(4-(5-(2-Nitrophenyl)-1,3,4-oxadiazol-2-yl)phenyl)-4H-chromen-4-one (***12***)

Solid; Yield 84%; m.p. 287–288 °C; ^1^H-NMR (MeOD-*d*_4_, 500 MHz): *δ* 8.15–8.06 (m, 6H), 7.90–7.70 (m, 3H), 7.32 (s, 1H), 7.15 (t, *J* = 7.0 Hz, 1H), 6.94 (d, *J* = 7.0 Hz, 1H), 6.86 (t, *J* = 7.0 Hz, 1H); ^13^C-NMR (125 MHz, DMSO-*d*_6_): *δ* 177.6, 164.5, 164.5, 163.5, 156.1, 146.8, 135.2, 135.1, 131.5, 130.2, 129.8, 128.2, 127.3, 127.3, 126.8, 126.8, 125.6, 125.2, 124.2, 123.6, 123.1, 116.0, 104.3; HR-EI-MS: *m*/*z* Calcd for C_23_H_13_N_3_O_5_, [M]^+^ 411.0855; found 411.0833.

### 4.15. 2-(4-(5-(4-Methoxyphenyl)-1,3,4-oxadiazol-2-yl)phenyl)-4H-chromen-4-one (***13***)

Solid; Yield 88%; m.p. 307–308 °C; ^1^H-NMR (DMSO-*d*_6_, 500 MHz): *δ* 8.08 (br. s, 4H), 7.75–7.70 (m, 3H), 7.33 (s, 1H), 7.18 (t, *J* = 7.0 Hz, 1H), 7.01 (d, *J* = 8.5 Hz, 2H), 6.94 (d, *J* = 7.5 Hz, 1H), 6.88 (t, *J* = 7.5 Hz, 1H), 3.80 (s, 3H); ^13^C-NMR (125 MHz, DMSO-*d*_6_): *δ* 177.4, 164.4, 164.4, 163.5, 160.5, 156.1, 135.1, 130.3, 129.0, 127.2, 127.2, 126.8, 126.8, 125.6, 125.2, 123.8, 123.2, 116.0, 115.8, 115.8, 114.6, 114.6, 104.4, 56.1; HR-EI-MS: *m*/*z* Calcd for C_24_H_16_N_2_O_4_, [M]^+^ 396.1110; found 396.1095.

### 4.16. 2-(4-(5-(3-Methoxyphenyl)-1,3,4-oxadiazol-2-yl)phenyl)-4H-chromen-4-one (***14***)

Solid; Yield 84%; m.p. 299–300 °C; ^1^H-NMR (MeOD-*d*_4_, 500 MHz): *δ* 8.11 (d, *J* = 7.0 Hz, 2H), 8.03 (d, *J* = 7.0 Hz, 2H), 7.72 (d, *J* = 6.0 Hz, 1H), 7.60 (s, 1H), 7.36–7.32 (m, 2H), 7.24–7.20 (m, 2H), 6.98 (d, *J* = 7.0 Hz, 1H), 6.95–6.93 (m, 2H), 3.92 (s, 3H); ^13^C-NMR (125 MHz, DMSO-*d*_6_): *δ* 177.5, 164.3, 164.3, 163.5, 161.0, 156.0, 135.0, 130.2, 130.1, 130.0, 127.2, 127.2, 126.6, 126.6, 125.6, 125.2, 123.7, 123.2, 119.6, 116.0, 114.2, 111.2, 104.3, 55.4; HR-EI-MS: *m*/*z* Calcd for C_24_H_16_N_2_O_4_, [M]^+^ 396.1110; found 396.1093.

### 4.17. 2-(4-(5-(2-Methoxyphenyl)-1,3,4-oxadiazol-2-yl)phenyl)-4H-chromen-4-one (***15***)

Solid; Yield 84%; m.p. 286–287 °C; ^1^H-NMR (DMSO-*d*_6_, 500 MHz): *δ* 8.64 (s, 1H), 8.05–8.00 (m, 4H), 7.72 (d, *J* = 7.5 Hz, 1H), 7.53 (d, *J* = 7.0 Hz, 1H), 7.34 (s, 1H), 7.30 (t, *J* = 7.0 Hz, 1H), 7.15 (t, *J* = 7.0 Hz, 1H), 6.95–6.90 (m, 4H) 3.94 (s, 3H); ^13^C-NMR (125 MHz, DMSO-*d*_6_): *δ* 177.3, 164.3, 163.5, 162.5, 157.2, 156.0, 135.1, 130.2, 130.0, 129.6, 127.2, 127.2, 126.4, 126.4, 125.1, 125.6, 123.5, 123.1, 121.3, 116.5, 116.0, 112.5, 104.2, 56.0; HR-EI-MS: *m*/*z* Calcd for C_24_H_16_N_2_O_4_, [M]^+^ 396.1109; found 396.1090.

### 4.18. 2-(4-(5-(Pyridin-2-yl)-1,3,4-oxadiazol-2-yl)phenyl)-4H-chromen-4-one (***16***)

Solid; Yield 80%; m.p. 270–271 °C; ^1^H-NMR (MeOD-*d*_4_, 500 MHz): *δ* 8.30 (d, *J* = 7.0 Hz, 1H), 8.10–8.02 (m, 3H), 7.99–7.96 (m, 2H), 7.90 (t, *J* = 7.0 Hz, 1H), 7.70 (d, *J* = 7.0 Hz, 1H), 7.42 (d, *J* = 7.0 Hz, 1H), 7.24–7.20 (m, 2H), 6.96–6.92 (m, 2H); ^13^C-NMR (125 MHz, DMSO-*d*_6_): *δ* 177.2, 164.3, 164.3, 163.3, 157.2, 156.0, 149.1, 137.1, 135.0, 130.1, 127.2, 127.2, 126.4, 126.4, 125.6, 125.1, 124.4, 124.0, 123.5, 123.2, 116.0, 104.2; HR-EI-MS: *m*/*z* Calcd for C_22_H_13_N_3_O_3_, [M]^+^ 367.0957; found 367.0941.

### 4.19. 2-(4-(5-(Pyridin-3-yl)-1,3,4-oxadiazol-2-yl)phenyl)-4H-chromen-4-one (***17***)

Solid; Yield 82%; m.p. 280–281 °C; ^1^H-NMR (MeOD-*d*_4_, 500 MHz): *δ* 8.58 (d, *J* = 5.0 Hz, 1H), 8.44–8.40 (m, 2H), 8.05–8.00 (m, 4H), 7.75 (d, *J* = 6.0 Hz, 1H), 7.52 (d, *J* = 6.0 Hz, 1H), 7.23–7.20 (m, 2H), 6.94–6.90 (m, 2H); ^13^C-NMR (125 MHz, DMSO-*d*_6_): *δ* 177.3, 164.3, 164.3, 163.3, 156.0, 152.6, 147.8, 135.0, 134.0, 130.1, 127.2, 127.2, 126.4, 126.4, 125.6, 125.1, 124.3, 124.0, 123.5, 123.2, 116.0, 104.2; HR-EI-MS: *m*/*z* Calcd for C_24_H_16_N_2_O_4_, [M]^+^ 367.0957; found 367.0942.

### 4.20. 2-(4-(5-(Pyridin-4-yl)-1,3,4-oxadiazol-2-yl)phenyl)-4H-chromen-4-one (***18***)

Solid; Yield 84%; m.p. 288–289 °C; ^1^H-NMR (MeOD-*d*_4_, 500 MHz): *δ* 8.60 (d, *J* = 6.0 Hz, 2H), 8.04 (d, *J* = 6.0 Hz, 2H), 7.98 (d, *J* = 8.0 Hz, 2H), 7.84 (d, *J* = 6.0 Hz, 2H), 7.70 (dd, *J* = 7.0 Hz, 1.5 Hz, 1H), 7.22 (s, 1H), 7.18 (t, *J* = 7.0 Hz, 1H), 6.96–6.94 (m, 2H);^13^C-NMR (125 MHz, DMSO-*d*_6_): *δ* 177.4, 164.2, 164.2, 163.2, 156.1, 149.7, 149.7, 143.5, 135.1, 130.2, 127.2, 127.2, 126.4, 126.4, 125.4, 125.1, 123.6, 123.2, 121.1, 121.1, 116.0, 104.2; HR-EI-MS: *m*/*z* Calcd for C_24_H_16_N_2_O_4_, [M]^+^ 367.0957; found 367.0931.

### 4.21. 2-(4-(5-Phenyl-1,3,4-oxadiazol-2-yl)phenyl)-4H-chromen-4-one (***19***)

Solid; Yield 80%; m.p. 280–281 °C; ^1^H-NMR (DMSO-*d*_6_, 500 MHz): *δ* 8.39 (s, 1H), 8.8–803 (m, 6H), 7.72 (s, 1H), 7.23 (t, *J* = 7.0 Hz, 1H), 7.22–7.18 (m, 2H), 7.09 (d, *J* = 7.0 Hz, 1H), 6.94 (d, *J* = 7.0 Hz, 1H), 6.90 (t, *J* = 7.0 Hz, 1H), 6.80 (d, *J* = 7.0 Hz, 1H); ^13^C-NMR (125 MHz, DMSO-*d*_6_): *δ* 177.2, 164.1, 164.1, 163.2, 156.0, 135.0, 130.1, 129.0, 129.0, 128.5, 127.3, 127.3, 127.1, 127.1, 126.3, 126.3, 125.4, 123.4, 123.1, 121.3, 116.0, 112.5, 104.2; HR-EI-MS: *m*/*z* Calcd for C_23_H_14_N_2_O_3_, [M]^+^ 366.1004; found 366.0990.

### 4.22. β-Glucuronidase Assay

*β*-Glucuronidase activity was determined in accordance to method used by Taha et al. [43,44] by measuring absorbance at 405 nm of the *p*-nitrophenol formed substrate by a spectrophotometric method. The total reaction volume was 250 µLs. Reaction mixtures containing 5 µL of test compound solution, 185 µL of 0.1M acetate buffer and 10 µL of enzyme solution were incubated for 30 min at 37 °C. At 405 nm the plates were recorded on a multiplate reader (SpectaMax, Molecular Devices, Sunnyvale, CA, USA) after the addition of 50 µL of 0.4 mM *p*-nitrophenyl-*β*-d-glucuronide. Experiments were performed in triplicate [45]. To avoid precipitation, compound concentrations were decreased, and the volume of reaction was increased (200 µL). Precipitation probability was less thus addition of detergents was not needed.

### 4.23. Molecular Docking Studies Assay

In silico binding interactions of synthesized derivatives against human-*β*-glucuronidase were inspected using the Autodock Vina software [46]. The X-ray crystal structure of human-*β*-glucuronidase (PDB ID: 1bhg) was retrieved from the Protein Data Bank [http://www.rcsb.org/]. Water molecules, ligands and Chain-B were removed from the downloaded PDB file by means of Discovery Studio Visualizer [47]. Two-dimensional structures of synthesized compounds were sketched and their energies were optimized using Chembio3D Ultra software (Version; 14.0.0.117) (Cambridge Soft, PerkinElmer Inc., Boston, MA, USA). Both receptor and ligand files were changed into pdbqt format by adding charges “q” and autodock type “t” using Autodock Tools (ADT) [48]. Other parameters contained within configurational file were defined as follow; Grid Box of size 19 × × 19 Å centered at X = 81.25, Y = 85.15 and Z = 87. 35 so that the active site [49] is entirely enclosed, maximum number of modes were set 50, energy difference between finest and poorest binding mode was set 4 Kcal/mol and exhaustiveness = 32. Conformations with lowest free energy of binding were evaluated and visualized using Discovery Studio Visualizer.

## Figures and Tables

**Table 1:** Chromen-4-one based oxadiazole derivatives **1**–**19**.

S. No	R	IC_50_ ± SEM ^a^	S. No	R	IC_50_ ±SEM ^a^
**1**	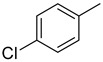	2.6 ± 0.1	**11**	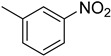	26.1 ± 0.5
**2**	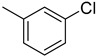	5.1 ± 0.2	**12**	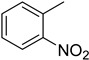	17.2 ± 0.4
**3**	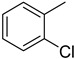	2.1 ± 0.1	**13**	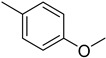	32.20 ± 0.6
**4**	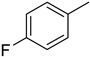	1.1 ± 0.05	**14**	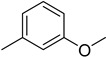	39.5 ± 0.7
**5**	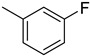	3.8 ± 0.2	**15**	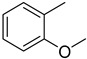	20.1 ± 0.5
**6**	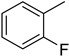	0.8 ± 0.1	**16**	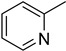	14.9 ± 0.4
**7**	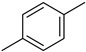	9.4 ± 0.3	**17**	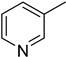	34.7 ± 0.6
**8**	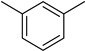	13.0 ± 0.3	**18**	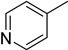	24.5 ± 0.5
**9**	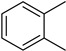	8.6 ± 0.3	**19**	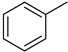	42.3 ± 0.8
**10**	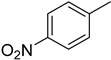	21.3 ± 0.4	d-saccharic acid 1,4 lactone ^c^		48.1 ± 1.2

^a^ standard error mean; ^c^ standard drug.
